# Supplemental treatment of rheumatoid arthritis with natural milk antibodies against enteromicrobes and their toxins: results of an open-labelled pilot study

**DOI:** 10.1186/1475-2891-10-2

**Published:** 2011-01-06

**Authors:** Kou Katayama, Takeo Matsuno, Takaki Waritani, Kuniaki Terato, Hiroshi Shionoya

**Affiliations:** 1 Katayama Orthopedic Rheumatology Clinic, Toyooka 13-4-5-17, Asahikawa, Japan; 2 Department of Orthopedic Surgery, Asahikawa Medical College, Asahikawa, Japan; 3 Chondrex Inc., Redmond, Washington, USA; 4 Asama Chemical Co. Ltd, Chuo-ku, Tokyo, Japan

## Abstract

**Background:**

Environmental factors, particularly commensal bacteria in the gastrointestinal tract, may be involved in the pathogenesis of rheumatoid arthritis (RA). The aim of this study was to evaluate whether natural milk antibodies against a wide spectrum of pathogenic enteromicobes and their toxins modify the disease activity in RA.

**Methods:**

Twenty patients with RA, whose disease activity was uncontrolled by authentic medications due to drug resistance, complications and/or risk factors were treated for 3 months with an oral administration of a whey protein concentrate (WPC) containing high levels of natural milk antibodies. Eighteen background-matched RA patients, not supplemented with milk antibody adjunct, were used as controls.

**Results:**

Statistically significant reduction of arthritis symptoms and improvement of intestinal disorders were observed only in the test group: effective in 8 (44%), possibly effective in 2 (12%) and not effective in 8 (44%) of 18 patients treated (2 patients withdrew) based on an *ad hoc *"evaluation point", the sum of variables that are improved more than 20% among the 8 core variables used for the American College of Rheumatology (ACR) response criteria. This disease modifying effect of the WPC disappeared upon cessation of treatment, but was reappeared upon reintroduction of it. Importantly, 7 of 8 non-responders carry DR15 haplotype (DRB1-1501 and 1502), whereas only 1 of 7 responders was DR15 positive (risk ratio: 6.1). Furthermore, the pre-clinical serum anti-LPS and anti-type II collagen antibody levels in the responders were higher or tended to be higher than those in the non-responders, suggesting that there are 2 sub-types of RA based on an interaction between gastrointestinal pathogens and MHC class II haplotypes.

**Conclusions:**

The natural milk antibody preparation containing high levels antibodies against pathogenic enteromicrobes and their toxins seems to be effective in a certain RA subset, and deserves more attention as a potential adjunct in the treatment of RA.

**Trial Registration Number:**

UMIN000003128

## Background

The disease causative factor of rheumatoid arthritis (RA) remains unknown regardless of extensive studies on candidate antigens[1-5] and disease susceptibility [6,7]. Recently, some consideration has been given to environmental factors, particularly commensal bacteria in the gastrointestinal (GI) tract [8,9]. For example, it has been shown that bacterial cell wall components, such as enterobacterial common antigens [10] and peptidoglycan-polysaccharide polymers, can induce arthritis [11,12] and uveitis [13] in experimental animals. GI bacteria and their toxins such as lipopolysaccharides (LPS: gram-negative bacteria cell wall components) apparently contribute to the development and exacerbation of autoimmune diseases in experimental models such as autoimmune thyroiditis in rats [14] and autoimmune hemolytic anemia (AIHA) in mice [15,16].

In clincal studies, it has also been suggested that commensal bacteria may play a pathogenic role in patients with RA. Aoki *et al*. reported that some patients with RA were sensitized to enterobacterial common antigens (35 and 38 kDa outer membrane protein) [10]. Van der Heijden and coworkers reported that degradation products of bacterial cell walls and nucleic acids were found in RA joints [17]. Imbalance of intestinal bacteria has even been suggested as a possible etiopathogenic or aggravating factor in RA based on the observation that modulation of the intestinal bacterial flora by a vegetarian diet was associated with clinical improvement [18-20]. Unfortunately, these observations were not acknowledged by researchers in the fields of immunology and rheumatology, because of the difficulty of handling and analyzing intestinal bacteria. However, Vaahtovuo *et al*. [21] recently reported that *Bifidobacteria*, bacteria of the *Bacteroides-Porphyromonas-Prevotella *group, *Bacteroides fragilis *subgroup, and *Eubacterium rectale-Clostridium coccoides *group were significantly less numerous in early RA than in controls as determined by flow cytometry analysis of 16 S rRNA hybridized and DNA-stained fecal bacteria.

Based on our [4,5,22,23] and other previous studies [24], it has been hypothesized that the increased permeability of the GI mucosa relating to a lowered immune function of gut associated lymphoid tissues (GALT) could modulate rheumatoid disease activity [25]. For example, excess amounts of bacteria toxins absorbed from the GI mucosa may directly stimulate the release of pro-inflammatory cytokines such as tumour necrosis factor (TNF), interleukin-1 (IL-1) [26], and high mobility group box-1 (HMGB1) protein [27], which could exacerbate inflammatory reactions [23,28,29], but also systemically affect the host's immune system for prolonged periods as non-specific immunostimulants.

The GI bacterial balance is modulated by interactions between pathogenic and non-pathogenic bacteria and by the host's immune function. Therefore, it is rational to consider how to alter or normalize intestinal environmental conditions by foods rather than antibiotics, since it is apparent that nutritional components apparently affect the intestinal bacterial flora population. In this aspect, we focused on natural milk antibodies, which recognize a wide spectrum of pathogenic entromicrobes and their toxins. Accordingly, we prepared a whey protein concentrate (WPC), which contains high levels of bioactive natural antibodies [30], from normal cow milk instead of colostrums using special conditions to avoid heat denaturation of immunoglobulins. This WPC was tested in elderly volunteers, and the test results clearly indicated that bioactive natural milk antibody preparation is capable of modulating intestinal bacterial flora, e.g. decreasing the population of *E. coli *and *Clostridium perfringens *(formally known *Clostridium welchii*) by 50 and 80% respectively in the feces, whereas increasing the population of *Lactbacilli *3-fold (Iwatsuki eta al: manuscript submitted). Based on these observations, in this study, we evaluated the disease modifying effect of this WPC in patients with RA. The results in this open labelled interventional study could shed light on the eventual involvement of enteromicrobes and their toxins in RA, prior to an eventual large-scale double blind controlled study.

## Methods

### Patients

This study (the trial registration number: UMIN000003128) was approved by the ethical committee of Katayama Orthopedic Rheumatology Clinic, and a written informed consent was obtained from all patients before performing any study procedures according to the Declaration of Helsinki. Thirty-eight voluntary patients with RA attending the Katayama Orthopedic Rheumatology Clinic and fulfilling the ACR diagnostic criteria of RA [31] were enrolled in this quasi-randomized study based on problems with authentic medications due to drug resistance, complications and/or risk factors. All patients continuously received the current treatments through this test period. The first twenty patients meeting the selection criteria received a whey protein product containing active antibodies in addition to their current medication (test group). The next 18 background-matched patients visiting the clinic were used as controls without administration of this supplement (control group). Eighteen out of 20 patients in the test group completed the study: 1 man and 17 women, average age 59.7 (range 31-80) years and average disease duration of 114.5 (range 3-360) months. Reasons for inclusion were resistance to concomitant drug therapy (5 patients), drug allergies (4), or complications and/or risk factors (9), such as interstitial pneumonitis (1), bilateral severe knee flexion contracture (1), cancer chemotherapy (2), breastfeeding (1), leucocytopenia (1), chronic heart failure (1), severe osteoporosis (1) and previous pneumonia (1). Mean prednisolone dose in the test group was 2.64 mg/day and mean 28 joint count disease activity score using ESR (DAS28-ESR) was 5.64 at the entry.

The eighteen background-matched control patients consisted of 1 man and 17 women, average age 62.8 (range 44-88) years and average disease duration of 88.1 (range 9-336) months. Mean prednisolone dose in this group was 2.72 mg/day, and mean DAS28-ESR at entry was 5.64. The basic pre-clinical data of patients in these 2 groups are shown in Table 1.

**Table 1:** Comparison of baseline clinical demographics of test and control groups

	Control Group	Test Group	P
Categories	(N = 18)	(N = 18)	
Clinical assessment at entry			
Age (Years)	62.8 ± 11.4	59.7 ± 14.9	NS^†^
RA duration (Months)	88.1 ± 76.2	114.5 ± 92.6	NS^†^
DAS28-ESR	5.64 ± 0.86	5.64 ± 1.37	NS^†^
TJC	12.3 ± 6.1	15.9 ± 11.7	NS^†^
SJC	12.6 ± 6.2	13.9 ± 7.8	NS^†^
ESR (mm/hr)	46.7 ± 27.0	48.4 ± 32.9	NS^†^
CRP (mg/dl)	2.5 ± 2.4	2.5 ± 2.8	NS^†^
RF (IU/ml)	143 ± 102	213 ± 292	NS^†^

Medication			
PSL (mg/day)	2.72 ± 2.29	2.64 ± 2.94	NS^†^
PSL (No.)	13	11	NS^§^
DMARDs (No.)	16	13	NS^§^
NSAIDs (No.)	14	11	NS^§^

Ochi's Classification			
LES	3 (17%)	4 (22%)	NS^§^
MES	12 (66%)	10 (56%)	NS^§^
MUD	3 (17%)	4 (22%)	NS^§^

Complications and risk factor			
Complication (No.)	9	9	NS^§^
Drug resistance (No.)	7	5	NS^§^
Drug allergy (No.)	2	4	NS^§^

Gastrointestinal Disorders			
Constipation (No.)	12	12	NS^§^
Diarrhea (No.)	3	2	NS^§^
None (No.)	3	4	NS^§^

Data is shown as the mean ± standard deviation.

TJC: tender joint count, SJC: swollen joint count, RF: rheumatoid factor, PSL: Prednisolone, LES: least erosive subset, MES: more erosive subset, MUD: mutilating disease, NS: Not significant.

†: Determined by Mann Whitney *U*-test. §: Determined by Chi square test.

### Administration of milk antibody

In the test group, one 10 g package of whey protein concentrate containing natural milk antibodies (Bonyuno Chikara^®^) with water was added to the concurrent treatment daily after breakfast for 3 months, whereas control patients did not get this food supplement.

In 5 patients (ID 198, 3188, 3709, 3240 and 4119), who responded to this milk antibody treatment as described later, the treatment was repeated after cessation to assess the eventual effect of recall treatment. In addition, 1 patient (ID3240) volunteered for the 3rd time treatment.

### Whey protein

A whey protein product, Bonyuno Chikara^®^, was supplied by Asama Chemicals Inc. Tokyo, Japan. This product was prepared from normal cow milk instead of colostrums using special conditions to avoid heat denaturation of immunoglobulins, and has been shown to contains relatively high levels of active antibodies against at least 33 strains of pathogenic bacteria [30]. One 10 g packet contains 6 g of whey protein concentrate contains 240 mg natural immunoglobulins, equivalent to 500-600 ml of raw milk, and 3 g of fructooligosaccharide, 0.8 g of milk calcium, and 0.2 g of cellulose.

### Clinical assessments

All patients in the test group were evaluated every month during the 3-month treatment period, whereas patients in the control group were evaluated before and after 3 months using the ACR response criteria with 7 core set variables: acute phase reactants (C-reactive protein: CRP or erythrocyte sedimentation rate: ESR), 0-66 swollen joint count (SJC), 0-68 tender joint count (TJC), modified Health Assessment Questionnaire (mHAQ), patient's and physician's global assessment of disease activity by visual analogue scale (VAS), and patient's pain assessment by VAS [32], and the European League Against Rheumatism (EULAR) response criteria using DAS28-ESR [33].

However, the ACR response criteria were not considered to be appropriate for evaluating the indirect disease modifying effects of milk antibodies, which do not possess direct anti-inflammatory or analgesic actions. Therefore, the effect of milk antibody treatment was evaluated by using an *ad hoc *"Evaluation Point", which was set up for the descriptive purposes of this study. For this analysis, the 7 core set variables used in the ACR response criteria were divided into 8 variables by separating the acute phase reactants into 2 variables, CRP and ESR, to evenly emphasize the importance of these independent marker values. The sum of the variables, which gained more than 20% improvement, was defined as an evaluation point. Evaluation points of 3 out of 8 and over were considered to be effective and defined as responders, whereas evaluation points of 2 and under were considered to be not effective and defined as non-responders. In addition, individual patients were further evaluated for features not related to arthritis but to general health, such as GI status, appetite, weight gain, activities of daily living (ADL), anemia, fatigue, sleep and daily physical feeling, and required dosage of steroid or NSAID.

### Biological serum markers

Serum TNF and IL-6 levels were determined using ELISA kits for human TNF-α/TNSF1A and human IL-6 (R & D Systems, Minneapolis, MN, USA). Serum antibody levels against human, bovine and chicken type II collagen, and LPS from *E. coli *O26, O55 and O111 (Sigma-Aldrich, St. Louis, MO, USA) were assayed by ELISA as previously reported [4]. Briefly, ELISA plates were coated with antigen (5 μg/ml) dissolved in phosphate buffer (μ = 0.4), pH 7.6. Antigen non-coated wells were used as a blank to determine background values of individual samples. A full strength buffered normal goat serum, pH 8.0, was used for blocking and sample dilution [4]. The secondary antibodies, biotin-conjugated goat anti-human IgG and IgA antibodies (Sigma, USA), were diluted in 2% normal goat serum, whereas streptavidin-peroxidase was diluted in 2% milk casein hydrolysate dissolved in 0.1 M Tris-HCl buffer, pH 7.5. All serum samples were diluted 1:100 and incubated with antigens at room temperature for 2 hours. Colour was developed using tetramethylbenzidine (TMB), and optical density (OD) values of antigen non-coated wells (blank values) of individual samples were subtracted from the OD values in antigen-coated wells.

For the anti-LPS antibody assay, affinity-purified bovine IgG anti-*E. coli *O111 LPS antibody was used as a reference to compare OD values in the same plate. For detection of bovine anti-LPS antibody, biotinylated goat anti-bovine IgG antibody (Jackson ImmunoResearch, West Grove, PA, USA) was used. Antibody concentrations in human serum samples are expressed as μg/ml.

### HLA typing

HLA types were analyzed using peripheral blood cells collected from individual patients by rssop method at HLA Laboratory, Kyoto, Japan.

### Statistical analysis

Data is expressed as the mean ± standard deviation except if otherwise indicated. For statistical evaluation, the paired Student's t-test was used to determine the significance of differences before and after treatment. The Mann-Whitney U-test was used for comparisons between the control and test groups, and the responder and non-responder groups. Chi-square test with Fisher's exact probability test was used for analysis of incidence and prevalence data. Results are shown using P values with 5% level of significance if not otherwise stated. The relationship of HLA haplotypes and therapeutic effect of milk antibodies was analyzed by a prospective cohort analysis using an equation, Relative risk = [a/(a+b)]/[c(c+d)], whereas a: number of patients with DR15+ (non-responder), b: DR15+ (responder), c: DR15- (non-responder), and d: DR15- (responder).

## Results

### Clinical demographics of patients in test and control groups

Baseline clinical demographics of the test and control groups were similar (Table 1). Based on the number of milk packages returned at the monthly controls, compliance with taking the whey protein was high, and only 2 of 20 patients withdrew due to disliking of the milk flavour. One patient (ID3188) reported aggravated constipation during the second treatment period, but otherwise no adverse effects were reported.

### Evaluation of disease modifying effect of milk antibody treatment

Apparent improvement of disease marker values such as CRP and SJC was observed as early as 1 month in several patients in the test group and lasted until the end of the study, where such changes were not seen in the control group. After 3-months of treatment, all patients were evaluated by 3 methods, the evaluation point, ACR, and EULAR response criteria (Table 2), in addition to general health assessment by both the patient and physician (see remarks in Table 2).

**Table 2:** Evaluation of therapeutic effect of milk antibody treatment in 18 patients in test group.

Patient ID	Eval Point^1)^	ACR^2)^ Criteria	DAS28-ESR				Remarks of improvements		DRB1- Allele	
					
			Pre	Post	Δ	EULAR^3)^	GI^4)^	General condition^5)^		
Responders										
3709	8	20	6.01	3.47	2.54	Moderate	C (+)	Fatigue, Anemia, Weight loss	0101	0405
3188	8	20	6.31	4.82	1.49	Moderate	C (+)	ADL	0405	0404
4119	6	50	4.61	3.39	1.22	Moderate	(/)	Range of motion	0901	1406
198	6	<20	8.02	7.13	0.89	No	C (+)	Fatigue, Anemia	0803	1401
5284	5	<20	4.32	3.07	1.25	Good	C (+)	Unhealthy feeling	0410	1502
10572	3	<20	6.12	6.08	0.04	No	C (+)	Appetite, Sleep	1302	1406
1316	3	<20	4.34	4.21	0.13	No	C (+)	Fatigue	ND	ND
3240	3	<20	5.29	4.63	0.66	Moderate	D (+)	Unhealthy feeling	0405	1202

*Ave*	*5.3 ± 2.1*		*5.63 ± 1.26*	*4.66 ± 1.43*	*0.97 ± 0.87*		*(7/7)*			
*Pre vs. Post*					*P < 0.01*		*P < 0.01*			

Partial responders										
169	2	<20	5.26	6.22	-0.96	No	(/)	Fatigue, Intra-articular effusion	0901	-
511	1	<20	8.15	7.85	0.30	No	D (+)	Range of motion	0405	1101

*Ave*	*1.5 ± 0.7*		*6.75 ± 2.10*	*7.04 ± 1.15*	*-0.33 ± 0.89*		*(1/1)*			

Non-Responders										
9291	2	<20	4.70	5.04	-0.34	No	C (+)	-	0803	1501
3007	1	<20	4.44	5.85	-1.41	No	(/)	-	1101	1502
2264	1	<20	8.00	7.86	0.14	No	C (+)	-	0405	-
721	1	<20	6.33	6.29	-0.04	No	C (+)	-	0405	1501
7785	0	<20	6.22	7.58	-1.36	No	C (-)	Edema at lower thigh	0901	1501
2110	0	<20	ND	ND	ND	ND	C (-)	-	0405	1502
8861	0	<20	4.01	5.68	-1.67	No	C (-)	-	0901	1501
10164	0	<20	4.45	4.94	-0.49	No	(/)	-	0405	1501

*Ave*	*0.6 ± 0.7*		*5.45 ± 1.44*	*6.18 ± 1.15*	*-0.73 ± 0.74*		*(3/6)*			
Pre vs. Post					NS		*NS*			

*Responder vs. Non-Responder*			NS	P < 0.05						

1) Number of categories among 8 categories gained more than 20% improvement. 2) American College of Rheumatology, 3) European League Against Rheumatism response criteria using DAS28-ESR. 4) Gastrointestinal disorder, C: Constipation, D: Diarrhea, (+): improved, (-): not improved, (/): no GI disorder. 5) ADL: activities of daily living.

ND: Not determined, NS: Not significant.

By the evaluation point analysis, milk antibody treatment was considered to be effective (evaluation point: 3 and more) in 8 of 18 (44.4%) patients but not effective (evaluation point: fewer than 2) in 10 of 18 (55.6%) patients in the test group. On the other hand, no improvement was observed in18 control patients during this 3-months test period, except 1 patient scored 3 (data not shown). Among 10 patients with on evaluation point less than 2 in the test group, 2 patients (ID169 and 511) with mutilating disease and diagnosed as most functionally severe subtype of RA by Ochi's classification [34] were classified as partial responders, because clinical and general health parameters were apparently improved as shown in " Remarks" in Table 2.

Among the 8 patients classified as responders by the evaluation point, 2 patients improved by 20% (ACR20) and 1 patient by 50% (ACR50) according to the ACR response criteria, whereas DAS28-ESR improvement was good in 1 patient, moderate in 4 patients, and poor in 3 patients by EULAR response criteria as shown in Table 2. None of the 2 partial responders, 8 non-responders and 18 control patients showed improvement by either ACR or EULAR evaluation.

General health assessment was performed by both the patient and physician for fatigue, anemia, mobility, ADL, appetite, and GI status such as constipation and diarrhea (see Remarks in Table 2). Notably, constipation or diarrhea was observed in 12 and 3 patients out of 18 patients, respectively, in the control group, and 12 and 2 patients out of 18 patients in the test group (Table 1). In the test group, these symptoms were alleviated in 11 of 14 patients: 7 of 7 responders, 1 of 1 (ID 511) partial responder, and even in 3 of 6 patients (ID 721, 2264 & 9291) in the non-responder group, but in none of the 15 patients in the control group (P < 0.01).

### Effect of milk antibody treatment on clinical marker values

**Figure 1:**
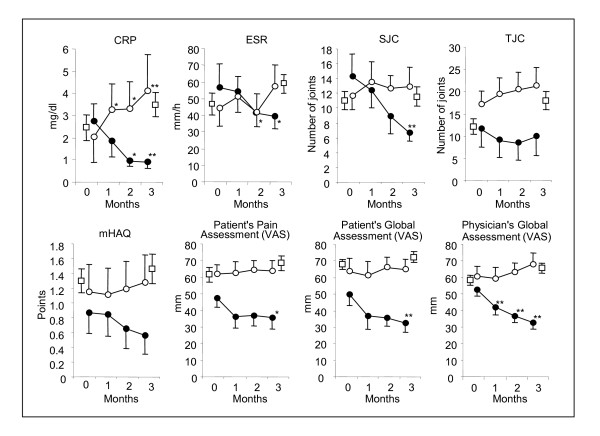
**Comparison of clinical marker value changes between responder and non-responder groups**. Eight variables used for ACR response criteria were determined every month, and the average values for responder (<< ocirc >>) and non-responder (<< ccirc>>) group were plotted. The average values for non-treated controls (<< osq >>) at 0 and 3 month were also shown for comparison. Bar: Average ± SE, *: P < 0.05, **: P < 0.01

The temporal changes of the 8 core variables during the treatment with milk antibody are shown in Figure 1 to compare the trends between responders and non-responders in the test group and non-treated patients in the control group. In the control group, ESR (p < 0.05) and TJC (p < 0.01) increased due to the persistence of the disease during the 3-month period. In contrast, the mean values of all 8 variables declined or tended to decline in responders of the test group, whereas all these values tended not to change or increase in non-responders. The most remarkable and steady improvement was observed in CRP and SJC, although significant improvement was also observed in the other 4 variables, ESR, patient's pain assessment, and patient's and physician's global health assessments by VAS at the end of the 3-month treatment. Associated with these trends, the mean of DAS28 values in the control group and the non-responder group increased or tended to increase from 5.64 ± 0.86 to 6.29 ± 0.94 (p < 0.05) and 5.45 ± 1.44 to 6.18 ± 1.15 (NS), whereas DAS28 values in the responder group decreased from 5.63 ± 1.26 to 4.66 ± 1.43 (p < 0.01) (Table 2).

### Confirmation of disease modifying effect of milk antibody treatment

**Figure 2:**
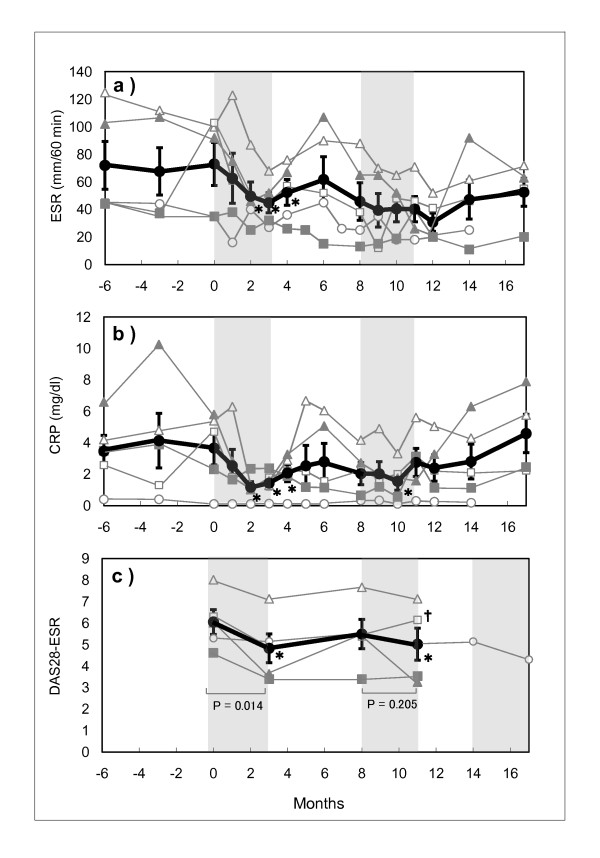
**Transitional changes of CRP (a), ESR (b) and DAS28-ESR (c) before, during and after the repeating treatment with milk antibody with 4 months resting period**. Thin lines with <<otri>>: Patient ID198, <<osq>>: ID3188, <<ocirc>>: ID3240, <<ctri>>: ID3709, <<csq>>: ID4119, and Heavy line with <<ccirc>>: Average ± SE, *: P < 0.05 (compared to the values at the starting point of milk antibody treatment), cross mark: Constipation worsened during secondary treatment with milk antibodies (ID3188), Shadow area: Treatment period with milk antibodies. NOTE: CRP and ESR values of 5 patients who received the second treatment among the 8 responders (Figure 1) were plotted in Figure 2(a) (b) and (c). Due to abnormal CRP and ESR values in one patient (ID4119) at the 3rd month of the second treatment, the average values of both CRP and ESR at the end of the second treatment seem to increase slightly.

Five patients from the responder group (ID198, 3188, 3709, 3240 and 4119) volunteered an additional 3-months of treatment after a 4-month washout period. CRP and ESR levels had decreased and remained at low levels during the first treatment period, but gradually increased and reached the pre-treatment levels approximately 2 months after discontinuation (Figure 2a and 2b). During the second treatment period, CRP and ESR levels decreased again, but increased after termination of the treatment. Similarly, TJC and SJC values tended to improve during the second treatment (the mean of TJC value decreased from 12.4 to 9.6, and the mean of SJC value from 13.2 to 8.8). DAS28-ESR also tended to decrease during the second treatment (Figure 2c), except in 1 patient (ID 3188) whose constipation worsened during the second treatment period.

### Differences between responders and non-responders to milk antibody treatment: possible contribution of DR15 Haplotype

**Figure 3:**
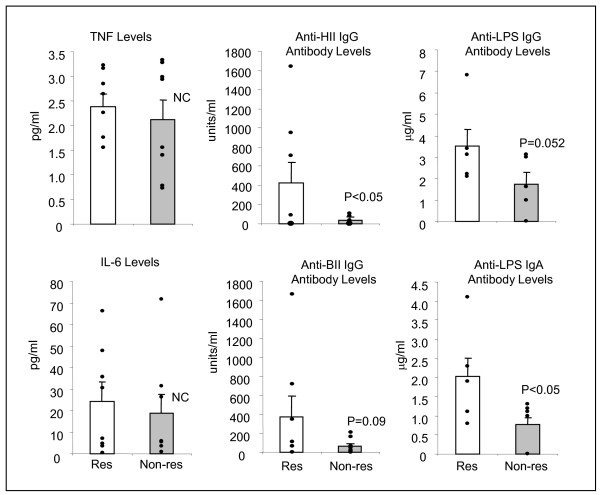
**Comparison of pre-clinical serum biological markers between responders and non-responders**. <<ccirc>>: individual patients, Open column: Responder, Shaded column: Non-responder, Bar: Average ± SE.

In order to search for the fundamental difference between the responders and the non-responders, the pre-clinical biological marker values before starting this treatment were compared (Figure 3). There were no significant differences in serum TNF-α and IL-6 levels, which represent the inflammatory reactions. However, anti-human type II collagen antibody levels in the responder group were higher than those in the non-responder group (P < 0.05). Similarly, serum IgG and IgA anti-LPS antibody levels tended to be higher or were higher in the responder group compared to the non-responder group (IgG: P = 0.052, IgA: P < 0.05) as shown in Figure 3.

Notably, only 1 of 7 responders who had been tested for their HLA type was positive for the DR15 (DRB1-1501 & 1502) haplotype, whereas 7 of 8 non-responders carried DR15 (P < 0.05). The risk factor value of DR15, calculated by a prospective cohort analysis, was 6.1 for non-responsiveness (p < 0.01 by Fisher's exact test), whereas the risk factor value of DR4 (DRB1-0404 & 0405) was 0.88 (Table 2).

## Discussion

Various milk antibody products produced from immune and normal colostrums have been previously tested as food supplements for the treatment of infectious diarrhea in newborn farm animals, human infants, and patients with AIDS (see reveiw [35]). Based on our previous obsevations that milk antibodies may prevent the overgrowth of pathogenic bacteria and subsequently reduce the bacteria toxin production, we have studied the effect of milk antibodies on the disease activity in patients with RA. In the present study, we found that milk antibody treatment was associated with clinical improvement in 10 out of 18 patients with RA, which was uncontrollable by current therapeutics due to drug resistance, complications and risk factors. In the responder group, CRP and ESR values were significantly decreased and remained at low levels during and even 1 month after the termination of treatment, suggesting perhaps a disease modifying rather than placebo effect. More importantly, use of natural antibodies was associated with alleviation of GI disorders in most of the cases, 11 of 14 patients with GI disorders in the test group. Especially, all 7 of 7 responders with GI disorder displayed the improvment of GI disorders, which was associated with improvement of arthritis and biological markers such as CRP and ESR. However, in non-responder group, no apparent improvement of arthritis symptom was observed in 3 patients regardless of improvement of GI disorders, indicating that other internal or external factors may be involved in the pathogenesis of RA or simply the dose of milk antibodies (240 mg as immunoglobulin) used in this study was not sufficient.

Since most of the patients enrolled in this study were elderly (60 ± 15 years old), it is likely that their intestinal bacteria flora balance was subject to overgrowth of certain strains of potentially pathogenic bacteria due to the lowered immune function at GALT associated with immunosenescence, which may contribute in their non-responsiveness to kefir, an immunostimulant, as shown in rats [36]. In young adult rats, kefir enhanced immune responses to intraduodenumly inoculated cholera toxin, but kefir was not effective in elderly rats. Based on the evidence that the majority of patients suffering from gastrointestinal disorders and related diseases are elderly, milk antibodies which directly affect the intestinal bacteria is considered to be more superior than kefir. This speculation is supported by our previous studies on the effect of milk antibody treatment on intestinal bacteria flora in 47 elderly volunteers, which clearly showed that milk antibody treatment significantly reduced the population of *E. Coli, Clostridium perfringens, Clostridium difficile, Clostridium *subcluster XIVa OTU369 and *Bacteroides *OTU853, whereas it increased the population of *Lactobacillus, Bacterides fragilis*, genera of *Bacteroides *and *Prevotella*, *Clostridium *subcluster XIVa OTU995, *Bacteroides *OTU366, and an unidentified species of OTU443 (Iwatsuki et al, submitted). Importantly, it has been suggested that bovine immunoglobulins were partially resistant to proteolytic digestion in the human stomach and small intestine [37]. Indeed, approximately 800 μg (0.24%) of 320 mg of bovine immunoglobulin was recovered in feces from these volunteers. These observations coincide and are in agreement with previous observations that vegetarian diet altered the intestinal bacterial flora in patients with RA [18,19] and the change in bacteria flora could affect the outcome of disease activity.

It has been reported that translocation of bacterial cells, cell components and toxins is increased by cold [38], heat [39], psychological stress [40], non-steroidal anti-inflammatory drugs [41], surgery [42] and constipation [43]. Khalif *et al*. reported that *E. coli *and *S. aureus *population in the feces increased in patients with chronic constipation, and their mucosal permeability for heterologous proteins was increased 30-fold compared to normal values. As a consequence, serum antibody levels to *E. coli *and *S. aureus *were significantly increased in these patients [43]. Via such mechanisms, milk antibodies may indirectly reduce translocation of bacterial toxins [42] and pathogen-associated moleculules with pro-inflammatory and adjuvant effects [44], which might affect the disease activity in RA.

In this study, we found that pre-clinical serum IgA and IgG antibody levels to *E. coli *LPS (IgA: P < 0.05, IgG: P = 0.052) and IgG anti-bovine type II collagen antibodies (P < 0.09) were higher or tended to be higher in the responders than the non-responders, indicating that responders might have higher mucosal permeability as suggested by Khalif *et al *[43]. In this aspect, it will be important to notice the reports showing that some patients with RA were sensitized by enterobacterial common antigens (35 and 38 kDa outer membrane protein) [10], and degradation products of bacterial cell walls and nucleic acids were found in RA joints [17].

These phenomena might be linked to genetic backgrounds as we observed in this study that there was an association between DR 15 negativity and responsiveness to milk antibodies. HLA DR15 positive patients who did not respond to the milk antibody intervention had low antibody titer to both LPS and type II collagen. Although it is not clear how DR15 contributes to the non-responsiveness to milk antibody treatment, there are potentially 2 subtypes of RA depending on an interaction between gastrointestinal pathogens and MHC class II haplotypes. It is possible that environmental factors are involved in the ethiopathogenesis of autoimmune diseases, and toll-like receptors (TLRs) that recognize molecular patterns displayed by microorganisms including LPS may play a key role in activation of the innate and adaptive immune systems [44].

## Conclusions

Whey protein concentrate containing active form of natural milk antibodies seems to be a safely used food supplement which has a potential to modulate autoimmune mediated inflammatory reactions in a subset of patients with RA. It is concluded that this pilot study encourages future randomized controlled clinical trial to scientifically test this hypothesis.

## Competing interests

No sources of funding were used for this study. The whey protein product, "Bonyuno Chikara," used in this study was supplied by Asama Chemical Co. Ltd., Tokyo. Asama Chemical Co. Ltd., was involved in data collection and analysis, and the study design, but had no role in data interpretation, manuscript preparation, review, or the manuscript approval. Asama chemical Co. Ltd., owns the pending international patents for the use of natural milk antibodies, the main components of Bonyuno Chikara.

HS has served as a consultant for Asama Chemical Co. Ltd., received his salary, and initiated the series of studies on bioactive natural antibodies contained in milk, which was used for this trial. KK, KT and TW are independent, but have been collaborating with HS on the possible contribution of gastrointestinal disorders, which may be linked to overgrowth of pathogenic bacteria and their toxins, particularly LPS, in the pathogenesis of RA. TM is a collaborator with KK in clinical field for many years, but has no relation with neither Asama chemical nor KT, TW and HS.

## Authors' contributions

KK and TM designed and conducted the clinical study, collected and interpreted data, and wrote this report. KT and HS created and rationalized the basic concept of this study, contributed in the designing of this trial, interpreted the data, and wrote this report. TW analyzed and finalized the data for publication. The corresponding author had full access to all the data and final responsibility for the decision to submit the report for publication. All authors read and approved the final manuscript.
